# Remote Monitoring of Cardiac Implantable Electronic Devices in Very Elderly Patients: Advantages and Specific Problems

**DOI:** 10.3390/jcdd11070209

**Published:** 2024-07-03

**Authors:** Roberto Scacciavillani, Leonidas Koliastasis, Ioannis Doundoulakis, Sotirios Chiotis, Athanasios Kordalis, Maria Lucia Narducci, Sotiris Kotoulas, Gaetano Pinnacchio, Gianluigi Bencardino, Francesco Perna, Gianluca Comerci, Konstantinos A. Gatzoulis, Dimitris Tsiachris, Gemma Pelargonio

**Affiliations:** 1 Department of Cardiovascular & Pulmonary Sciences, Catholic University of the Sacred Heart, 00168 Rome, Italy; roberto.scacciavillani@gmail.com (R.S.); gemma.pelargonio@policlinicogemelli.it (G.P.); 2 First Department of Cardiology, National and Kapodistrian University, “Hippokration” Hospital, 11527 Athens, Greece; lkoliastasis@gmail.com (L.K.); sotirischg@gmail.com (S.C.); akordalis@gmail.com (A.K.); soter96@icloud.com (S.K.); kgatzoul@med.uoa.gr (K.A.G.); dtsiachirs1@gmail.com (D.T.); 3 Department of Cardiovascular Medicine, Fondazione Policlinico Universitario A. Gemelli IRCCS, 00168 Rome, Italy; marialucia.narducci@policlinicogemelli.it (M.L.N.); gaetano.pinnacchio@policlinicogemelli.it (G.P.); gianluigi.bencardino@policlinicogemelli.it (G.B.); francesco.perna@policlinicogemelli.it (F.P.); gianluca.comerci@policlinicogemelli.it (G.C.); 4 Cardiology Unit, Cardiothoracic Department, Azienda Ospedaliera Universitaria Santa Maria della Misericordia, 33100 Udine, Italy

**Keywords:** cardiac implantable electronic devices, CIED, remote monitoring, arrhythmic monitoring, hemodynamic monitoring

## Abstract

Cardiac implantable electronic devices (CIEDs) offer the benefit of remote monitoring and decision making and find particular applications in special populations such as the elderly. Less transportation, reduced costs, prompt diagnosis, a sense of security, and continuous real-time monitoring are the main advantages. On the other hand, less physician–patient interactions and the technology barrier in the elderly pose specific problems in remote monitoring. CIEDs nowadays are abundant and are mostly represented by rhythm control/monitoring devices, whereas hemodynamic remote monitoring devices are gaining popularity and are evolving and becoming refined. Future directions include the involvement of artificial intelligence, yet disparities of availability, lack of follow-up data, and insufficient patient education are still areas to be improved. This review aims to describe the role of CIED in the very elderly and highlight the merits and possible drawbacks.

## 1. Introduction

Cardiac implantable electronic devices (CIEDs) offer the opportunity for remote cardiac monitoring and decision making and may provide clinical benefits in terms of morbidity and mortality in a wide range of patients of all ages and different indications [1]. Due to the increased average life expectancy, the number of subjects with an implanted device continues to rise, in parallel with their mean age [2]. Remote monitoring (RM) is lately becoming an important tool to meet the necessity for regular check-ups of these numerous systems, and its widespread use has been recommended in recent consensus documents and fostered by the COVID-19 pandemic as a way to avoid or reduce patient visits [3,4]. Despite the widespread availability of modern RM technologies, their utilization remains low, particularly among individuals who would greatly benefit from them, such as the elderly [5]. In fact, comorbidities in the geriatric population significantly restrict patients’ lifestyles and can severely diminish their physical or mental independence; therefore, platforms for remote health monitoring could help in the management of this population [4]. Still, evidence regarding the clinical effectiveness of these devices and consequent RM in older adults is scarce and inconsistent, and randomized trials often do not accurately depict this subset of patients. Moreover, in this ever-growing subset of patients, RM faces specific challenges but could also produce significant benefits. This review focuses on the application of the various available CIEDs in the elderly and appraises the advantages and concerns associated with them.

## 2. Rhythm Remote Monitoring Systems Technical Aspects

RM relies on the collaboration of several essential components. Firstly, the patients occupy a central role in RM, with their cooperation and adherence being vital for realizing the associated clinical benefits. Then, there is the remote device clinic, with a multidisciplinary team of electrophysiologists, heart failure (HF) specialists, and dedicated technicians and nurses. Lastly, there are the home monitors and the RM platforms through which data can be accessed by the RM staff after recruitment. The main commercially available systems are represented by Merlin.net™ (Abbott, Chicago, IL, USA), Home Monitoring™ (Biotronik, Berlin, Germany), Latitude™ (Boston Scientific, Natick, MA, USA), CareLink™ (Medtronic, Minneapolis, MN, USA), and SmartView™ (MicroPort, Shanghai, China). They all have programmable alerts and provide the clinic with real-time electrograms of various durations. For the purpose of this review, we included all types of available devices with RM from these manufacturers (i.e., loop recorders, pacemakers, defibrillators, and cardiac resynchronization devices). The frequency of transmissions is programmable as well, except for Biotronik devices, which transmit every day. There are numerous kinds of alerts that depend on the device type and should be tailored to the patient. Below, we briefly identify device-integrity-related and clinical alerts [6].

**Figure 1:**
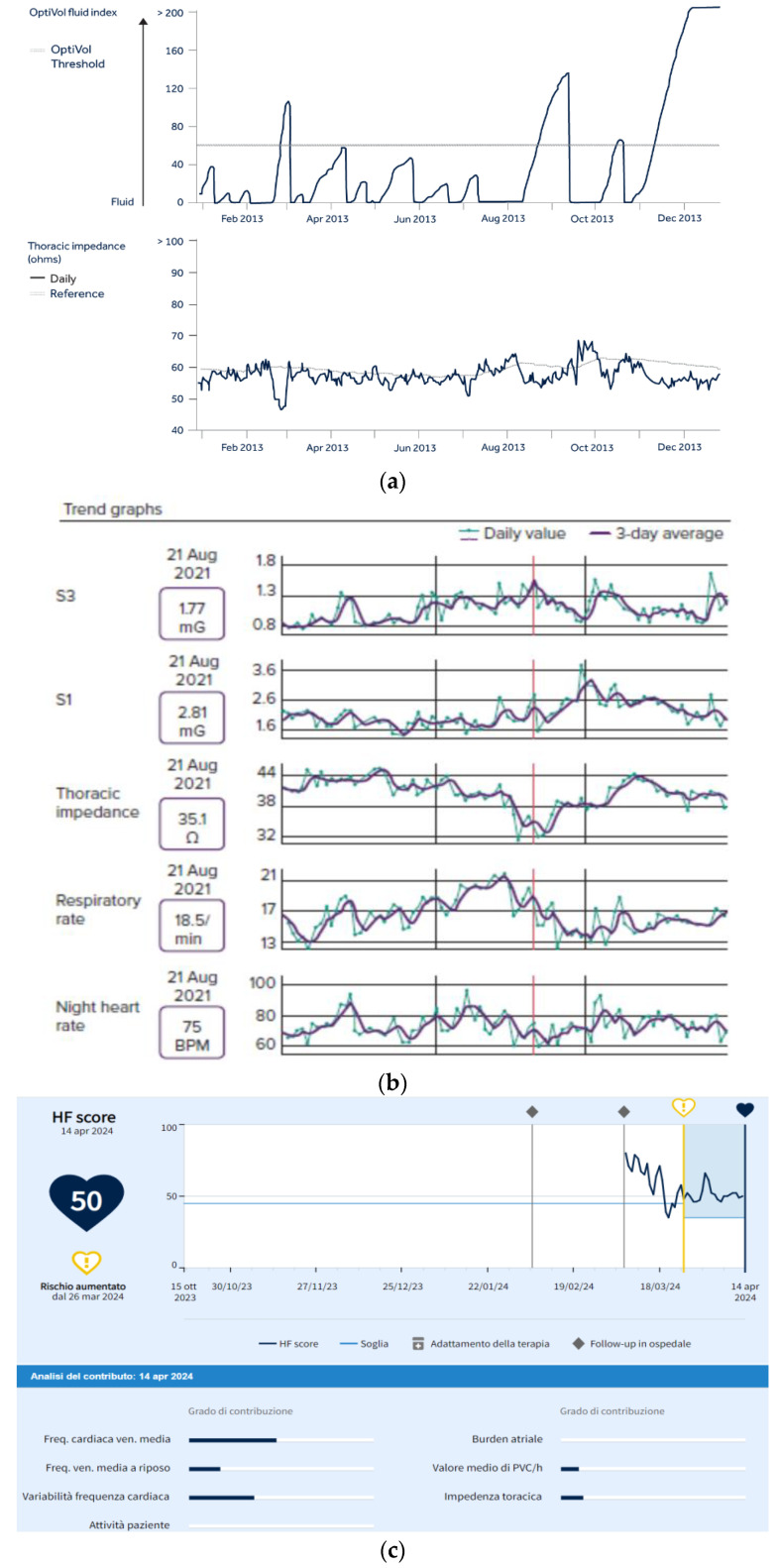
Screenshot from remote monitoring online platforms depicting heart failure management solutions: (**a**) Medtronic OptiVol™ fluid index reflecting the difference between the daily impedance and the reference impedance, adjusted for individual patient variation; (**b**) Boston Scientific HeartLogic™ algorithm relying on S3 and S1 heart sounds, thoracic impedance, respiratory rate and night heart rate; (**c**) Biotronik HeartInsight algorithm combining diurnal and nocturnal heart rates, ventricular extrasystoles, atrial tachyarrhythmia burden, heart rate variability, physical activity and thoracic impedance.

- Device-related alerts comprise end-of-service/low battery in all devices and lead to impedance, noise episodes, safety mode episodes, the pacing threshold being out of range (only in pacemakers (PM)), and implantable cardioverter defibrillators (ICD).
- Clinical alerts include brady and tachyarrhythmias in implantable loop recorders (ILR); the percentage of pacing, atrial high-rate episodes (AHREs), atrial fibrillation (AF) and ventricular high-rate episodes in PM and ICD; anti-tachycardia pacing (ATP) and/or shock in ICD recipients. Certain devices (mainly ICD and cardiac resynchronization therapy devices) also provide alerts on the HF status of the patient. Medtronic features OptiVol™ 2.0 Fluid Status Monitoring to track intrathoracic impedance changes over time and assess congestion to help predict hospitalizations [7,8]. The Boston Scientific HeartLogic™ algorithm relies on thoracic impedance, respiratory rate and patterns, heart rate, heart sounds, and patient activity to timely predict HF decompensation [9]. Biotronik developed another algorithm (HeartInsight) combining temporal trends of diurnal and nocturnal heart rates, ventricular extrasystoles, atrial tachyarrhythmia burden, heart rate variability, physical activity, and thoracic impedance, which are obtained to effectively predict HF hospitalizations [10]. Figure 1 displays an overview of the three HF interfaces.

## 3. Specific Remote Monitoring Related Issues in Elderly Patients

**Figure 2:**
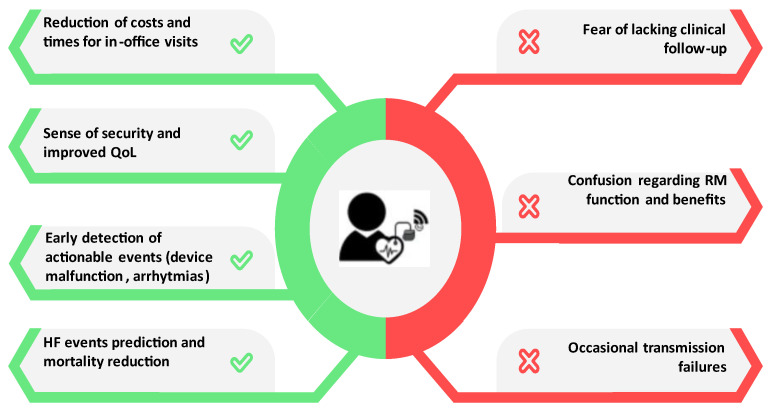
Benefits and obstacles of remote monitoring in very elderly patients. QoL: quality of life; HF: heart failure; and RM: remote monitoring.

Considering all the functions and the organization of RM, there are several subsequent obstacles in all patients that are more pronounced in the elderly (Figure 2). First, we must consider the psychological impact of a novel technology on the elderly population, who are usually reluctant to change the “status quo”. Patients with fewer interactions with healthcare providers may be afraid that they will lack appropriate clinical follow-up. A survey-based study reported that there are several reasons for old people preferring conventional in-office visits: encompassed interpersonal interactions with physicians with the possibility of posing inquiries, reliance on physicians and reassurance in consulting a cardiologist over relying solely on remote patient monitoring systems, proximity to the hospital for convenient travel, and instances of dissatisfaction with the remote patient monitoring system [11]. Some of them also expressed a preference for more frequent hospital visits exceeding once a year. There were also patients who remained indifferent to the choice; however, while recognizing the advantages of remote patient monitoring, they underscored the irreplaceable nature of hospital visits [11]. Moreover, the perceived lack of information at the moment of recruitment led to a failure to fully understand the benefits and functioning of RM [12]. Another aspect to consider is that transmitter handling can be cumbersome for elderly subjects, who frequently suffer from dementia, memory impairment, and/or hypoacusis. These frequent conditions could theoretically have a negative impact on RM handling, and there have been occasional reports of transmission failures in the elderly [13]. On the other hand, a significant proportion of patients also expressed a preference for continuous feedback through remote monitoring over the concept of “no news is good news”, as this could generate feelings of abandonment and anxiety [14].

In this regard, a further downside of missing out on one-on-one contact in this subset of patients with various comorbidities is the risk of overlooking a worsening of general clinical conditions due to this shift in care [14]. Communication with the patient and the family is, therefore, of great importance to stress the concept that RM is not a substitute for clinical follow-up, so the patients will neither feel insecure nor think that they can skip in-office visits. Consequently, providing the patient with periodic and comprehensible updates on the state of CIEDs, for example, via emails or other means of communication, may be psychologically and clinically valuable.

## 4. Main Advantages of Remote Monitoring in Elderly Subjects

Elderly patients often have reduced mobility, and RM monitoring diminishes the expenses for transportation for in-person visits. The necessity of accompanying the patient is abolished, and the workload of medical professionals is reduced [3,12,15]. Notably, especially after the COVID-19 era experience, RM reduces visits and thus the risk of sinfections and may be performed safely in frail patients. While we have previously stated that RM can have negative psychological effects on elderly people, observational studies reported satisfaction and acceptance among patients using RM, giving them a sense of security, even if not all of them had a complete understanding of the system [16,17]. However, these high levels of patient acceptance and satisfaction may be biased by the fact that only patients who were already participating in RM were included.

RM is also useful in detecting actionable events, such as atrial and ventricular arrhythmias. AF prevalence increases with age and is associated with worse outcomes. Stroke risk increases with age, and the medical treatment itself may lead to more complications in the elderly [18,19]. RM has been shown to reduce inappropriate ICD therapies via the early detection of fast AF with rapid ventricular response rates and T-wave oversensing issues in an observational prospective study on 1404 patients [20]. A randomized trial and a meta-analysis showed that the detection of AF episodes and its burden can prompt clinical intervention, thus possibly preventing stroke, shock therapy, and HF [21,22]. 

Patients on the whole spectrum of HF represent 1–2% of the adult population, and the incidence of HF progressively increases with age, reaching around 20% among people over 75 years [23]. The economic burden for health systems is massive; however, the most widely used remote diagnostic tools are still based on non-invasive, low-cost and low-sensitivity modalities, such as the electrocardiogram [24,25]. It is still unclear if the use of the algorithms based on single or multiple parameters described before may reduce HF hospital admissions and have mortality benefits. The initial negative results of the MORE-CARE multi-center randomized controlled trial (RCT) [26] are counterbalanced by the positive results of other RCTs, such as the MultiSENSE and the OptiLink HF, both for HF decompensation and cardiovascular death [9,27]. Recent meta-analyses confirmed this trend towards better outcomes when compared to standard care [28,29]. Therefore, it is reasonable to assume that the elderly population, which is exposed to the highest risk of HF hospitalization, is the one that could benefit most from RM. 

## 5. Implantable Hemodynamic Monitoring Devices

Implantable hemodynamic monitoring (IHM) has received a class IIb recommendation in the 2021 ESC Heart Failure guidelines; it is yet underused because of its invasive nature, high cost, and lack of clear benefits in hard endpoints. The concept of monitoring pulmonary arterial pressures is not new and has been studied for almost 30 years [30].

The IHM devices are based on the right chamber, pulmonary artery, and direct left atrial pressure real-time measurements so that the prompt modification of medical treatment may reduce the need for HF decompensation and hospitalization (Table 1). The rationale is that non-invasive approaches of volume (weight, echocardiographic measurements, biomarkers) fail to detect the developing decompensation in a timely manner and guide the treatment [31]. Research has shown that cardiac decompensation is not well predicted by right atrial pressure elevation or inferior vena cava alterations, whereas left ventricular filling pressures and their surrogates are better predictors [32]. Consequently, elevations in the intracardiac filling pressures may set an alarm for imminent decompensation, and the trend that is created from ambulatory IHM devices guides the physicians performing treatment towards modifying the diuretic treatment.

### 5.1. CardioMEMS™

The CardioMEMS™ HF system (Abbott, Chicago, IL, USA) consists of an implantable component and an external electronic monitoring unit. The implantable sensor is battery-free and wireless and is designed to measure changes in pulmonary artery pressures. It is 15 mm long and 3 mm wide, and it is implanted in a branch of the left pulmonary artery through right heart catheterization. The measurement process requires the external component (an antenna in the shape of a pillow) to be placed against the patient’s body, and information is uploaded into a secure monitoring system. The device is able to provide data on systolic, diastolic, and mean pulmonary pressure, pulmonary arterial waveform, and heart rate. It is of high importance that measurements have been validated with gold-standard right catheterization procedures [34,35]. The sensor is endothelialized into the pulmonary arterial wall and is compatible with other devices and magnetic resonance imaging.

For the CHAMPION RCT, daily artery pressure information was found to reduce the primary endpoint of hospitalization for HF significantly in the treatment group (n = 270) over 6 months (0.32 events/patient/6 months vs. 0.44 events/patient/6 months, *p* = 0002) [36]. The control group (no pressure monitoring, n = 280) experienced fewer changes in medication, which reflects the dynamic nature of HF diuretic treatment. Based on the baseline characteristics, these findings apply mostly to HF with reduced ejection fraction, and the results are consistent in 18-month long-term follow-up [37]. The GUIDE-HF trial, on the contrary, randomized 1000 patients into pulmonary pressure monitoring vs. the standard of care and resulted in no significant difference in the primary endpoint of all-cause mortality, HF hospitalization, or urgent HF visits (0.56 events/person-year vs. 0.64 events/person-year, HR: 0.88, 95%CI: 0.74–1.05, *p* = 0.16). However, it is notable that the pre-COVID-19 analysis showed a reduction in events in favor of CardioMEMS™ [38]. In the MONITOR-HF trial, 348 patients were randomized to either receive CardioMEMS or standard therapy for HF [39]. The primary outcome of the mean change in the Kansas City Quality of Life summary score at 12 months was +7.05 (95%CI: 2.77 to 11.33) vs. −0.08 (95% CI: −3.76 to 3.60), with *p* = 0.013 in favor of the treatment group with a reduction in HF hospitalizations or urgent HF visits. The non-randomized real-world studies COAST, MEMS-HF, the post-approval study, and the study by Desai et al. have all consistently demonstrated significantly reduced HF hospitalization rates for the CardioMEMS™ groups [33,40,41,42]. The device was also shown to be effective when used in patients receiving levosimendan, allowing personalization of the scheme and a reduction in the total costs [43,44].

### 5.2. Cordella™

The Cordella™ (Endotronix Inc., Chicago, IL, USA) device is a platform that consists of an implantable sensor, a wireless reading device, and peripherals (blood pressure, heart rate, weight, oxygen saturation). The sensor has not yet obtained a CE mark or FDA approval and is implanted into the left pulmonary artery and transmits data on mean pulmonary arterial pressure. The first-in-human SIRONA trial explored the device’s results in 15 patients and found the accuracy of measurements and safety [45]. Following this, the SIRONA II is ongoing and enrolled 70 patients to obtain a CE mark. The primary results support the safety and efficacy of the Cordella™ system regarding the primary endpoint of the accuracy of pressure measurements (equivalence bounds of mean pulmonary arterial pressure: −4.0 to 4.0mmHg) and 98% freedom from device-related complications [46]. The safety and accuracy of the device were consistent in the 12-month follow-up results [47]. The ongoing single-arm Proactive-HF trial is designed to assess the benefit of the personalized and proactive treatment of HF using the Cordella™ device in terms of performance/safety and clinical outcomes of HF hospitalization and mortality [48].

### 5.3. V-LAP

The V-LAP™ (Vectorious Medical Technologies, Tel-Aviv, Israel) is a leadless implantable monitoring system that is intended to detect and transmit remotely left atrial pressures. The device is a battery-free double-disk sensor and is implanted on both sides of the intraventricular septum. It is powered by an external reader and provides ambulatory measurements of the left atrial pressure and waveform. The VECTOR-HF trial enrolled 30 patients, and at 3 months of freedom, major adverse cardiac and neurological events were 97% and provided accuracy for the measured pressures compared to standard wedge pressure measurements (mean difference of −0.22  ±  4.92) [49]. The device lacks a CE mark or FDA approval, and there is no available research data on clinical endpoints.

### 5.4. Older Devices No Longer Available 

The first device of remote IHM was the Chronicle™ right ventricular pressure monitoring system Medtronic, Inc., Minneapolis, MN, USA) that resembled a pacemaker generator. A transvenous lead was fixed into the right ventricle and accompanied by an external reference device, which measured hemodynamic parameters. It was studied in the COMPASS-HF and REDUCE-HF RCTs and was proven to be safe but did not reduce clinical events [50,51].

The HeartPOD™ monitoring system (Abbott, formerly St. Jude Medical/Savacor, Inc., Chicago, IL, USA) comprised an implantable sensor that was placed through transeptal access to the left atrium and measured left atrial pressure and waveform, temperature and intracardiac electrocardiogram and had an external reader. The LAPTOP-HF RCT resulted in initially lower hospitalizations but was terminated due to high rates of procedural complications [52].

Remote monitoring via IHM devices in HF patients is promising and may provide better guidance on medical treatment and prevent hospitalization events. Increased costs, the possible need for future intervention in the same anatomic region, and the necessity of patient compliance and cooperation pose challenges. The careful selection of candidates is essential to unlock the full potential of the devices.

## 6. Future Directions

With constant technological advancements, RM will continue to evolve and play a key role in its transition to a tailored, patient-specific approach. In this perspective, several future improvements seem likely and even necessary. Remote programming will probably be the next step forward. Bidirectional communication between the device and the clinic may further reduce the need for inpatient visits and solve a range of device-related issues, such as sensitivity adjustments, threshold rise, and the fine-tuning of detection zones, with clear benefits for all patients, particularly the elderly. However, some limitations need to be considered, mostly related to cybersecurity and the lack of immediate feedback after reprogramming [53]. Another intriguing possibility is the exploitation of IHM data in association with CIED-derived information to create combined scores and platforms able to predict HF events more reliably. This may also lead to more impactful therapeutic refinements not only with pharmacological adjustments but also through HF device reprogramming [54].

Artificial intelligence and machine learning are nowadays a field of increasing interest and may be used to analyze the large amount of data obtained from RM to uncover patterns that precede critical events, such as ventricular arrhythmias, HF decompensations, and sudden cardiac death [55]. Substantial milestones have been reached in terms of diagnostics and therapeutics in the field of heart failure. The digitalization, combination, and analysis of multidimensional multimodal data and the creation of prediction models can improve the workflow and enhance the safety and effectiveness of the various treatments [56]. In that direction, a future upgrade could be represented by wearable digital health technologies and their integration into clinical practice and possibly also into the RM of patients with CIEDs. Standardization of transmissions, the patient trust-built, and managing the overwhelming amount of data all present upcoming major challenges that may could be overcome with the help of artificial intelligence [57].

## 7. Conclusions

The RM of CIEDs’ function has become the standard of care, and it is now recommended for all patients by professional societies, whereas RM for hemodynamic parameters is still evolving and being refined [1,3]. Disparities of availability, a perceived lack of clinical follow-up, and insufficient patient education are still areas that need further improvement to enhance patient adherence and their sense of security. However, the advantages in terms of reduced costs, time spent for in-office visits, device function, and clinical events seem to clearly outnumber these obstacles, and future refinements may bring additional improvements. These considerations are especially true in elderly subjects who, given their specific situation and additional risks, represent the population that could benefit the most from the implementation of RM strategies.

## Figures and Tables

**Table 1:** Overview of the remote implantable hemodynamic monitoring devices for heart failure.

Device	Implantation Site	Monitoring Parameters	Key Studies
CardioMEMS	Left pulmonary artery	PAP, heart rate	CHAMPION. GUIDE-HF, MONITOR-HF MEMS-HF COAST Post-approval study Desai et al. 2017 [33]
Cordella	Right pulmonary artery	PAR, blood pressure, heart rate, weight, SpO2	SIRONA, SIRONA-II, PROACTIVE-HF
V-LAP	Intraventricular septum	LAP	VECTOR-HF
Chronicle	Right ventricle	RVP, heart rate, temperature, estimated diastolic PAP	RECUCE-HF COMPASS-HF
HeartPOD	Left atrium	LAP, temperature, intracardiac ECG	LAPTOP-HF

LAP: left atrial pressure, RVP: right ventricular pressure, PAP: pulmonary arterial pressure, and SpO2: hemoglobin saturation of O2.

## Data Availability

Not applicable.
